# Mediterranean Diet Pattern: Potential Impact on the Different Altered Pathways Related to Cardiovascular Risk in Advanced Chronic Kidney Disease

**DOI:** 10.3390/nu16213739

**Published:** 2024-10-31

**Authors:** Jordi Rovira, María José Ramirez-Bajo, Elisenda Bañon-Maneus, Pedro Ventura-Aguiar, Marta Arias-Guillén, Barbara Romano-Andrioni, Raquel Ojeda, Ignacio Revuelta, Héctor García-Calderó, Joan Albert Barberà, Ana Paula Dantas, Maribel Diaz-Ricart, Fàtima Crispi, Juan Carlos García-Pagán, Josep M. Campistol, Fritz Diekmann

**Affiliations:** 1 Laboratori Experimental de Nefrologia i Trasplantament (LENIT), Institut d’Investigacions Biomètiques August Pi i Sunyer (IDIBAPS), 08027 Barcelona, Spain; mramire1@recerca.clinic.cat (M.J.R.-B.); ebanon@recerca.clinic.cat (E.B.-M.); pventura@clinic.cat (P.V.-A.); marias@clinic.cat (M.A.-G.); irevuelt@clinic.cat (I.R.); jmcampis@clinic.cat (J.M.C.); 2 Red de Investigación Cooperativa Orientada a Resultados en Salud (RICORS 2040), 28029 Madrid, Spain; 3 Department of Nephrology and Kidney Transplantation, Clínic’s Institute of Nephrology and Urology (ICNU), Hospital Clinic de Barcelona, Institut d’Investigacions Biomèdiques August Pi i Sunyer (IDIBAPS), Universitat de Barcelona, 08036 Barcelona, Spain; bromano@clinic.cat (B.R.-A.); raqojeda@gmail.com (R.O.); 4 Barcelona Hepatic Hemodynamic Laboratory, Liver Unit, Hospital Clínic_Clínic Barcelona, Institut de Investigacions Biomèdiques August Pi i Sunyer (IDIBAPS), Health Care Provider of the European Reference Network on Rare Liver Disorders (ERN-RareLiver), Department of Medicine and Health Sciences, University of Barcelona, CSUR_EVH, 08036 Barcelona, Spain; hgarcia@recerca.clinic.cat (H.G.-C.); jcgarcia@clinic.cat (J.C.G.-P.); 5 Centro de Investigación Biomédica en Red Enfermedades Hepáticas y Digestivas (CIBEREHD), 28200 Madrid, Spain; 6 Department of Pulmonary Medicine, Hospital Clínic, Institut d’Investigacions Biomèdiques August Pi i Sunyer (IDIBAPS), Universitat de Barcelona, 08036 Barcelona, Spain; jbarbera@clinic.cat; 7 Biomedical Research Networking Center on Respiratory Diseases (CIBERES), 30627 Madrid, Spain; 8 Cardiovascular Institute, Hospital Clinic de Barcelona, Institut d’Investigacions Biomèdiques August Pi i Sunyer (IDIBAPS), Universitat de Barcelona, 08007 Barcelona, Spain; adantas@recerca.clinic.cat; 9 Hematopathology, Centre Diagnòstic Biomèdic (CDB), Hospital Clinic de Barcelona, Institut d’Investigacions Biomèdiques August Pi i Sunyer (IDIBAPS), Universitat de Barcelona, 08007 Barcelona, Spain; mdiaz@clinic.cat; 10 Barcelona Endothelium Team (BET), 08036 Barcelona, Spain; 11 BCNatal|Fetal Medicine Research Center, Hospital Clínic and Hospital Sant Joan de Déu, Institut d’Investigacions Biomèdiques August Pi i Sunyer (IDIBAPS), Universitat de Barcelona, 08007 Barcelona, Spain; fcrispi@clinic.cat; 12 Centre for Biomedical Research on Rare Diseases (CIBER-ER), 28029 Madrid, Spain

**Keywords:** metabolomic and proteomic analysis, chronic kidney disease, cardiovascular disease

## Abstract

Background: Cardiovascular disease (CVD) remains the most common cause of mortality in chronic kidney disease (CKD) patients. Several studies suggest that the Mediterranean diet reduces the risk of CVD due to its influence on endothelial function, inflammation, lipid profile, and blood pressure. Integrating metabolomic and proteomic analyses of CKD could provide insights into the pathways involved in uremia-induced CVD and those pathways modifiable by the Mediterranean diet. Methods: We performed metabolomic and proteomic analyses on serum samples from 19 patients with advanced CKD (aCKD) and 27 healthy volunteers. The metabolites were quantified using four different approaches, based on their properties. Proteomic analysis was performed after depletion of seven abundant serum proteins (Albumin, IgG, antitrypsin, IgA, transferrin, haptoglobin, and fibrinogen). Integrative analysis was performed using MetaboAnalyst 4.0 and STRING 11.0 software to identify the dysregulated pathways and biomarkers. Results: A total of 135 metabolites and 75 proteins were differentially expressed in aCKD patients, compared to the controls. Pathway enrichment analysis showed significant alterations in the innate immune system pathways, including complement, coagulation, and neutrophil degranulation, along with disrupted linoleic acid and cholesterol metabolism. Additionally, certain key metabolites and proteins were altered in aCKD patients, such as glutathione peroxidase 3, carnitine, homocitrulline, 3-methylhistidine, and several amino acids and derivatives. Conclusions: Our findings reveal significant dysregulation of the serum metabolome and proteome in aCKD, particularly in those pathways associated with endothelial dysfunction and CVD. These results suggest that CVD prevention in CKD may benefit from a multifaceted approach, including dietary interventions such as the Mediterranean diet.

## 1. Introduction

Chronic kidney disease (CKD) is a complex disease with multiple etiologies, of which the most predominant are diabetes and hypertension. Patients with CKD have a 10–20-fold higher cardiovascular mortality rate compared to that of age-matched and sex-matched controls with normal renal function [1], with an annual mortality rate of 15–20% that is largely attributable to cardiovascular disease (CVD).

Endothelial dysfunction is well-characterized in CKD patients, regardless of the etiology, and is acknowledged as a major determinant for CVD when observed in this population [2]. In addition to CKD, these patients are also exposed to other non-traditional uremia-related CVD risk factors, including inflammation, increased oxidative stress, anemia, and abnormal calcium-phosphorus metabolism [3].

Several clinical trials have been conducted to analyze the impact of specific therapeutic agents, such as statins [4], erythropoiesis-stimulating agents [5], or AST-120 (Kremezin^®^; Kureha Chemical Industry Co. Ltd., Tokyo, Japan) [6], an oral, spherical activated carbon that can adsorb small-molecule uremic toxins, without identifying a clear benefit regarding the incidence of CVD or the progression to end-stage kidney disease (ESKD). Dietary modifications significantly impact patient outcomes and are postulated as an attractive complementary treatment alongside disease-specific drugs, without the constraints of secondary side effects. A growing body of evidence suggests that the Mediterranean dietary pattern (MD) exerts a significant impact on various physiological processes, including (i) a lipid-lowering effect, (ii) protection against oxidative stress, inflammation, and platelet aggregation, (iii) the inhibition of nutrient sensing pathways by specific amino acid restriction, and (iv) the gut microbiota-mediated production of metabolites that influence metabolic health. However, renal diets are quite restrictive regarding vegetable and bean consumption, due to their high concentrations of phosphorus and potassium. Nonetheless, given the benefits of DM on CVD, as well as the systematic demonstration that DM effectively preserves renal function and delays the progression of CKD [7,8,9,10,11,12], the KDOQI 2020 Clinical Practice Guideline for Nutrition in CKD [13], as well as the current KDIGO CKD 2024 guidelines [14], recommend considering the addition of a plant-based “Mediterranean-style” diet to lipid-modifying therapy to reduce CV risk. Kwon et al. have recently shown that MD is safe for patients with stage 3–4 CKD and may even contribute to preserving kidney function [15]. Interestingly, to date, it is not fully understood what the individual impact is of each food or nutrient found in the Mediterranean diet, in terms of its antioxidant and anti-inflammatory effects [16]. Several studies have identified many diet-associated metabolites [17,18,19]. Furthermore, a 67-metabolite signature has been developed to reflect adherence and the metabolic response to the MD, which is associated with reduced cardiovascular risk [20]. A better understanding of the altered pathways in advanced CKD (aCKD) patients, related to the risk of CVD, could help in the design of tailored Mediterranean diet schemes to optimize interventional strategies to reduce CVD risk in patients with CKD.

To explore such mechanisms, we performed an integrative metabolomic and proteomic analysis on serum samples from patients with chronic kidney disease (CKD) and healthy volunteers, with the aim of identifying significantly altered metabolic pathways that are relevant to the development of CVD and, in particular, endothelial dysfunction.

## 2. Material and Methods

### 2.1. Study Population

This was a cross-sectional study in which no intervention was employed. It was a nested case-control study within the project “Targeting endothelial dysfunction in highly prevalent diseases (PIE15/00027)”. Patients with an estimated glomerular filtration rate (eGFR), according to the CKD-EPI formula, of below 20 mL/min/1.73 m^2^ were prospectively included for the period between July 2016 and February 2018. Patients with essential arterial hypertension or dyslipidemia (defined by statin treatment) were included. Patients who presented any of the following concomitant pathologies associated with endothelial dysfunction were excluded from this study: kidney-related hereditary diseases, diabetes mellitus, malignancies, ischemic cardiovascular diseases, lung diseases, liver diseases, thyroid disorders, and active virus infection. Healthy volunteers were included as controls. The study protocol was approved by the local ethics committee on 5 October 2016 (HCB/2015/0585) and participating patients provided their written informed consent.

### 2.2. Epidemiological Characteristics

The following variables were collected at the time of patient inclusion; age, gender, systolic blood pressure, diastolic blood pressure, heart rate, respiratory rate, body mass index (BMI), and cardiovascular risk factors (dyslipidemia, hypertension, and chronic kidney disease). A blood test was performed to evaluate renal function (creatinine, eGFR), glucose, hemoglobin, Na, K, Ca, and P), inflammation (C-reactive protein), liver function (total bilirubin and direct bilirubin, AST, ALT, GGT, ALP), and lipid profile (total cholesterol, HDL, LDL, and triglycerides).

### 2.3. Sample Collection

Patient sera samples were collected at the Hospital Clinic de Barcelona. Blood was taken with a serum tube from an antecubital vein and was aliquoted within 1 h, then stored at −80 °C until metabolomic and proteomic analysis.

### 2.4. Metabolomic Analysis

Four different methods were employed for the extraction of hydrophobic lipids (including methanol extraction and the Folch method), the extraction of amino acids, and the extraction of polar metabolites in central carbon metabolism. Detailed methodology is provided as Supplementary Materials.

After metabolomic analyses, the lipidomic method, based on methanol extraction, provided semi-quantitative results for 204 lipids, the lipidomic method based on Folch extraction provided 119 lipids, the amino acid method provided semi-quantitative results for 49 amino acids and derivatives, and polar metabolites in the central carbon metabolism method provided 28 additional compounds. Thus, 400 unique metabolites were successfully quantified in the serum samples being analyzed and these can be found in Table S1. Some compounds can be determined in more than one analysis, after which the results with the highest level of confidence can be used.

### 2.5. Proteomic Analysis

Before the proteomic analysis, the seven most abundant serum proteins (albumin, IgG, antitrypsin, IgA, transferrin, haptoglobin, and fibrinogen) were depleted using a Human-7 Multiple Affinity Removal Spin (MARS) cartridge (Agilent Technologies, Santa Clara, CA, USA) to increase the number of identified proteins. Afterward, the samples were processed for tandem mass tag (TMT) before acquisition by nanoscale liquid chromatography coupled to mass spectrometry (nano LC-MS/MS) analysis, with an LTQ-Orbitran Velos Pro (Thermo Fisher Scientific, Waltham, MA, USA). Protein identification/quantification was performed using Proteome Discoverer software, version 1.4.0.288 (Thermo Fisher Scientific), by multidimensional protein identification technology. After proteomic analysis, a total of 273 proteins were identified in the samples analyzed (Table S2).

### 2.6. Statistical Analysis

#### 2.6.1. Data Pre-Processing

In the initial metabolomic and proteomic analyses, the readers were blinded to the patient’s status. For the statistical analyses, only those metabolites and proteins that were present in ≥70% of the samples in at least one group were considered. In addition, log base 2 transformation was applied to the protein data. Finally, the data were mean-centered and Pareto-scaled.

#### 2.6.2. Multivariate Statistical Analysis

Initially, a multivariate statistical approach was performed using Metaboanalyst 4.0 (http://www.metaboanalyst.ca/, accessed on 27 October 2024). The modeling process included the use of unsupervised methods, such as principal component analysis (PCA) and hierarchical clustering (HCA), and supervised methods, which included partial least-squares discriminant analysis (PLS-DA) and orthogonal projection to latent structures discriminant analysis (OPLS-DA).

#### 2.6.3. Univariate Statistical Analysis

For each metabolite or protein, a univariate test was performed. For the univariate tests, the data were not Pareto-scaled. Initially, the Control and aCKD samples were compared. A Kolmogorov–Smirnov test was carried out for each protein to check for distribution normality. Afterward, either a *t*-test or a Wilcoxon test was performed, depending on each protein’s distribution. In the case of a *t*-test, a test for equality of variance was performed prior to the analysis. The Benjamini–Hochberg method was used to adjust the *p*-values for multiple testing with the consideration of a 5% false discovery rate (FDR). The reported results included the means and standard deviations (SD) for each group, the fold change (FC), and the p and q (with p corrected for FDR) values. In addition, a ROC analysis was performed for each protein, and the area under the curve (AUC) and *p*-values are reported below.

#### 2.6.4. Pathway Analysis

The metabolites that were differentially presented in serum samples from aCKD patients were used to identify the differential pathways in an over-representation analysis (ORA), performed in Metaboanalyst using metabolite set enrichment analysis (MSEA).

Significantly different proteins identified with the univariate analysis were used for the pathway analyses, employing a method based on protein–protein interaction (PPI) networks and enrichment analysis from the Search Tool for the Retrieval of Interacting Genes/proteins (STRING) database (https://string-db.org/, accessed on 27 October 2024). In addition, an enrichment analysis was performed for each displayed network in STRING, testing a number of functional annotation spaces, including gene ontology (with three categories: biological process (GO-BP), cellular component (GO-CC), and molecular function (GO-MF)), the Kyoto Encyclopedia of Genes and Genomes (KEGG), Reactome, PFAM protein domains, and InterPro protein domains and features. The results of the enrichment are sorted according to their enrichment *p*-values, which were corrected for multiple testing using the Benjamini–Hochberg method.

The integrated pathway analysis was performed with the module Join Pathway Analysis from Metaboanalyst, combining metabolomics and gene expression (proteomics) studies conducted under the same experimental conditions. ORA, based on hypergenometrics analysis, was also chosen. The topology analysis evaluated whether a given protein or metabolite plays an important role in a biological response, based on its position within a pathway. Degree centrality was also used to measure the number of links that connect to a node (representing either a protein or metabolite) within a pathway.

## 3. Results

### 3.1. Epidemiological Characteristics

A total of 19 patients with advanced chronic kidney disease (aCKD) and 27 healthy volunteers were included in the study; the epidemiological characteristics are listed in Table 1. The aCKD patients were older than the volunteers in the control group (58.5 vs. 48.8 years; *p* = 0.0245). There were no differences between groups in terms of gender distribution and body mass index (BMI). The aCKD patients had a higher prevalence of dyslipidemia (57.9%) and hypertension (68.4%) and higher systolic blood pressure than the control group (132.3 vs. 109.1 mmHg) (*p* < 0.05). Triglycerides were increased in the aCKD patients, without differences being noted in the cholesterol levels.

Ten patients (52.6%) were classified as CKD stage G4, whereas nine patients (47.4%) were classified as stage G5. Regarding the etiology of primary renal disease, 6 (31.3%) patients had chronic hypertensive nephropathy, and 5 (26.3%) patients had IgA nephropathy, whereas the remaining 8 (42.1%) patients had CKD of unknown etiology.

The analysis of blood parameters revealed that sodium, phosphorous, glucose, and C-reactive protein levels were higher in aCKD patients, while the hemoglobin concentration was lower in aCKD patients (Table 1). Liver function was analyzed using AST, ALT, GGT, and ALP; all parameters remained in a non-pathological range, although the ALP value was higher in aCKD patients compared to the control group (Table 1).

### 3.2. Metabolomic Analysis

Of the total 383 metabolites considered, 135 of them were found to be differently presented in the serum from aCKD patients; of these, 54 metabolites had increased and 81 metabolites had decreased compared to the control serum samples (Table S3A and S3B, respectively). The analysis of lipids showed remarkably different results between aCKD patients and healthy volunteers. The aCKD patients had increased levels for 5 out of the 7 analyzed diglycerides (DG) and 6 out of the 25 triglycerides (TG). Furthermore, 29/35 lysophosphocholines (LPC), 1/35 lysophosphoethanolamines (LPE), 10/12 lysophoshoinositoles (LPI), 1/15 cholesteryl esters (ChoE), 6/23 sphingomyelin (SM), and 8/45 phosphocholine (PC) were observed to show reduced concentrations in aCKD patients.

### 3.3. Supervised Analysis

From the PLS-DA analysis, a clear separation between the control and aCKD patients could be observed. The best PLS-DA model included five components and showed a strong predictive ability (Accuracy = 1.0; R^2^ = 1.0 and Q^2^ = 0.81) (Figure S1A). The most important features contributing to class separation are shown in Table 2. The results from the OPLS-DA analysis showed similar results to those from the PLS-DA analysis (Table S4). Therefore, a significant model with strong predictive ability was obtained (Q^2^Y = 0.83, *p* < 0.001, 1 predictive + 3 orthogonal components), and the metabolites contributing to class separation can be identified in the corresponding S-plot (Figure S1B).

### 3.4. Pathway Analysis

**Figure 1:**
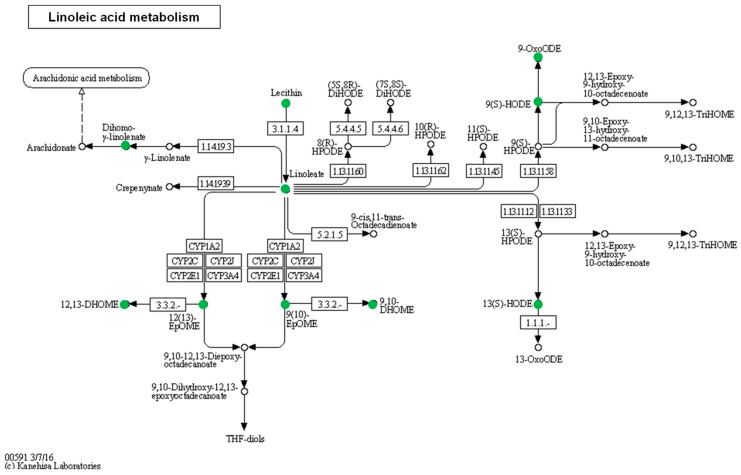
Linoleic acid metabolism, taken from the Kyoto Encyclopedia of Genes and Genomes (KEGG-hsa00591). The pathway analysis using metabolomic data showed seven downregulated metabolites (green dots). These metabolites were lecithin (phosphatidylcholine), linoleic acid, 12(13)-DiHOME, 12(13)EpOME, 9(10)-DiHOME, 9(10)EpOME, and 9-oxoODE. Furthermore, the integrative analysis showed two additional altered metabolites: 9-HODE/13-HODE and dihomo-γ-linoleic.

The ORA resulted in only one significantly enriched pathway, that for linoleic acid metabolism (Table S5). The aCKD patients had lower levels in 7 out of the 15 metabolites from this pathway (FDR = 8.62 × 10^−7^) (Figure 1 and Table 3).

### 3.5. Proteomic Analysis

A total of 164 proteins were considered, and 75 of them were found to be differentially expressed in the aCKD group compared to the control group. In addition, 38 proteins were upregulated compared to the control group, whereas 37 proteins were downregulated (Tables S6A and S6B, respectively). Our data confirmed the increased abundance of eight well-characterized uremic toxins, including beta-2-microglobulin, prostaglandin-H2 D-isomerase, and cystatin-C (Table S7).

### 3.6. Unsupervised Analysis

The PCA analysis and unsupervised clustering heatmaps showed a clear separation between the control and aCKD groups, without the presence of any outliers (Figure S2).

### 3.7. Supervised Analysis

From the PLS-DA analysis, a clear separation between the control and aCKD samples could be observed in the first component. The best PLS-DA model based on Q^2^ included two components and showed strong predictive ability (R^2^ = 0.97 and Q^2^ = 0.85). The most important features contributing to class separation are shown in Table 4. The results from the OPLS-DA analysis showed similar results to those from the PLS-DA analysis (Table S8). Therefore, a significant model with strong predictive ability was obtained (Q^2^Y = 0.90, *p* < 0.001) with 1 predictive + 1 orthogonal component, and the proteins contributing to class separation can be identified in the corresponding S-plot (Figure S3).

### 3.8. Pathway Analysis

**Figure 2:**
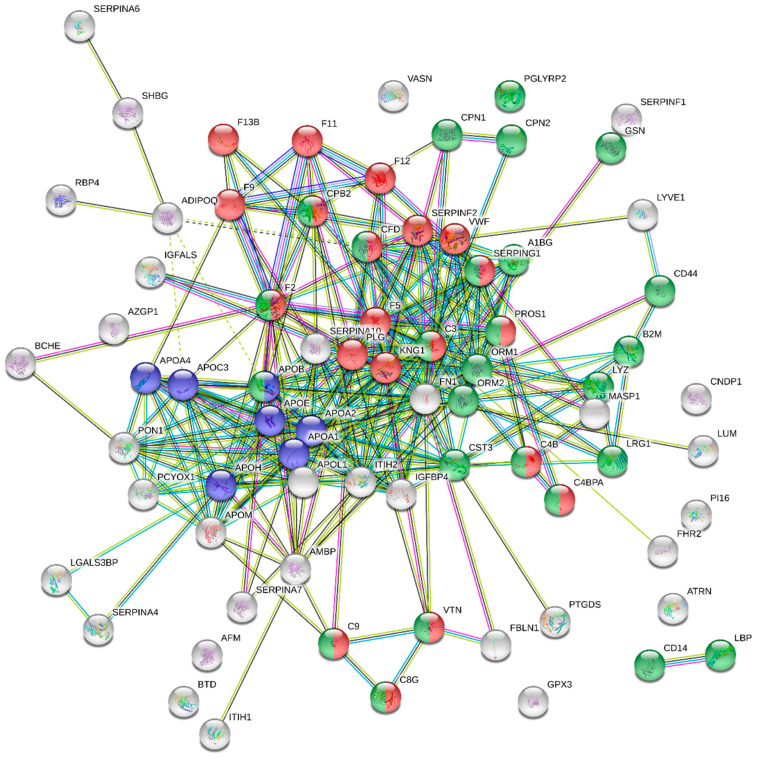
Protein–protein interaction network for the 75 statistically different proteins obtained using the STRING database. Here, the nodes represent proteins and edge interactions between proteins. The thickness of the edge indicates the degree of confidence prediction of the interaction. Only interactions with a high confidence score (>0.7) were considered. Proteins included in the complement and coagulation cascades appear in red, proteins from the innate immune system are shown in green, and proteins involved in cholesterol metabolism are in blue.

The inclusion of the 75 differentially expressed proteins in STRING software reflects 315 edges between 75 nodes, with a node degree of 8.4, a local clustering coefficient of 0.566, and a PPI enrichment *p*-value below 1.0 × 10^−16^ (Figure 2).

**Figure 3:**
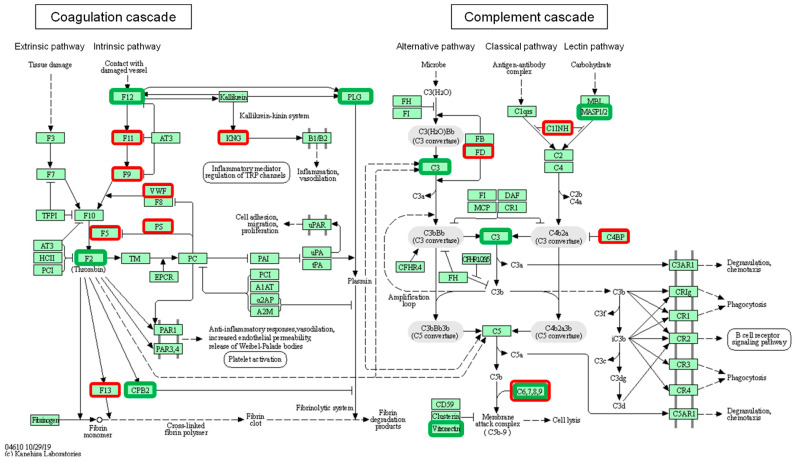
An illustration of the complement and coagulation cascades from KEGG-hsa04610. A total of 21 (out of 78) proteins were significantly enriched. Proteins that showed significantly lower and higher concentrations in aCKD are shown in green and red, respectively. The downregulated proteins were C3, C8G, CPB2, F12, F2, PLG, SERPINF2, VTN, and MASP1, whereas the upregulated proteins were C4B, C4BPA, C9, CFD, F11, F13B, F5, F9, LNG1, PROS1, SERPING1, and VWF.

The enrichment analysis showed that the complement and coagulation cascades (KEGG, 21 of 80 proteins, FDR = 3.15 × 10^−24^) (Figure 3 and Table 5), innate immune system (Reactome 26 of 1012 proteins, FDR = 1.023 × 10^−13^) (Table 6 and Figure S4), and cholesterol metabolism (KEGG, 7 of 48 proteins, FDR = 4.67 × 10^−8^) (Table 7 and Figure S5) were highly altered in aCKD patients. All the pathways that were altered are presented in Table S9.

In addition, the enrichment analysis classified all proteins according to three categories from the Gene Ontology resource: biological process (GO-BP), cellular component (GO-CC), and molecular function (GO-MF). The altered proteins found in aCKD patients are involved in the regulation of proteolysis (32 proteins, FDR = 4.05 × 10^−22^), in the inflammatory response (21 proteins, FDR = 3.20 × 10^−17^), and in the response to stress (46 proteins, FDR = 8.82 × 10^−16^), among other biological processes (Table S10). In terms of the cellular component, 72 out of 75 of the altered proteins in aCKD (96%) are located in the extracellular region (FDR = 1.35 × 10^−57^). Most of the proteins are found in the lumen of vesicles or the secretory granules (Table S11). Regarding molecular function, most of the altered proteins have enzyme regulator/inhibitor activity. These proteins mainly participate in peptidase, endopeptidase, or carboxypeptidase reactions (Table S12).

### 3.9. Integrated Pathway Analysis

The inclusion of the differentially expressed metabolites and proteins in the integrative analysis increased the statistical impact of several pathways that include proteins and metabolites (Table 8 and Table S13). Although the more significantly altered pathways in aCKD patients remained the complement and coagulation cascades, linoleic acid metabolism, and cholesterol metabolism, other pathways that also reached the level of statistical differences included fat digestion and absorption, protein digestion and absorption, the biosynthesis of unsaturated fatty acids, and beta-alanine metabolism.

### 3.10. Other Metabolite and Protein Biomarkers

**Figure 4:**
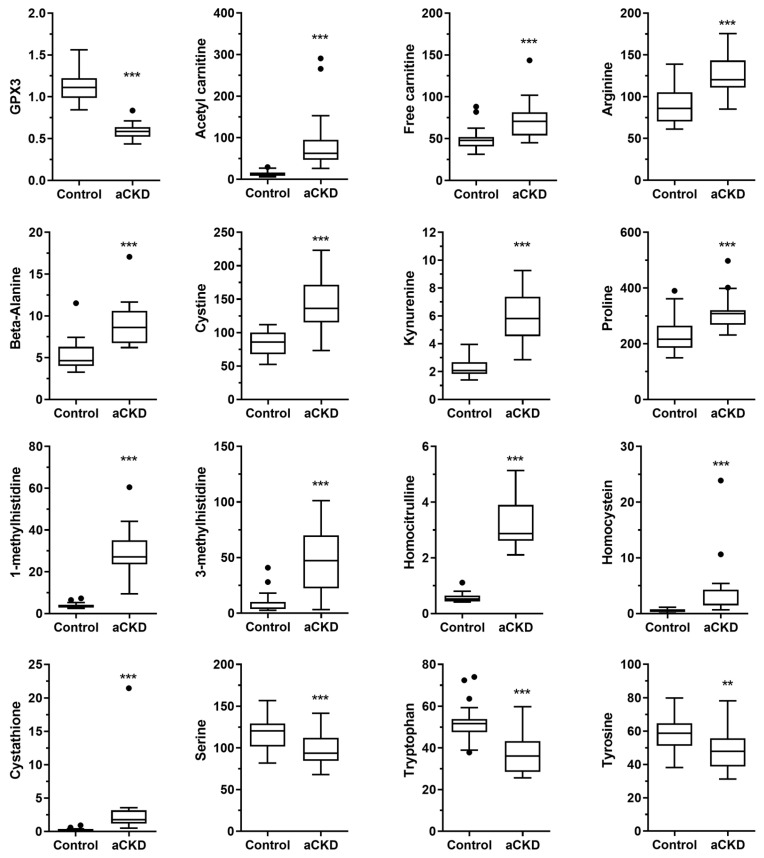
Quantification of the altered metabolites and proteins in aCKD patients. The Mann–Whitney test was performed. * Significantly different when compared to the control group (** *p* < 0.01; *** *p* < 0.001).

We analyzed several metabolites and proteins that are considered to be TOP biomarkers; however, they were not involved in the enriched pathways described above (Figure 4). The enzyme involved in the detoxification of hydrogen peroxide, glutathione peroxidase 3 (GPX3), was reduced in aCKD patients. Acetyl-carnitine and free carnitine were increased in the aCKD patients. Several amino acids and derivates (arginine, beta-alanine, cystine, kynurenine, proline, 1-methylhistidine, 3-methylhistidine, homocitrulline, homocysteine, and cystathionine) were increased in aCKD patients, whereas three (serine, tryptophan, and tyrosine) were reduced.

## 4. Discussion

**Figure 5:**
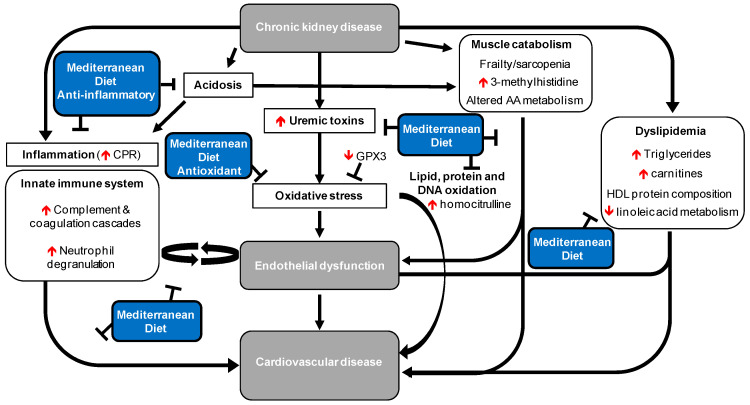
Altered pathways in aCKD patients, related to the risk of CVD development. The metabolites, proteins, and pathways identified in this study are accompanied by the symbols ⇅, indicating increases or reductions in aCKD patients. Mediterranean diet interventions that could reduce CVD risk in patients with CKD are shown in blue boxes.

The metabolomic and proteomic analysis of serum samples showed that numerous pathways are disrupted in aCKD patients, all of which exert direct or indirect effects on the cardiovascular system. We propose an interaction model of these altered pathways to elucidate the impact of aCKD on the development of CVD (Figure 5). Additionally, based on the literature, we identify potential pathways that could be modulated by a Mediterranean diet to reduce the risk of CVD in aCKD patients.

A Mediterranean diet pattern is built around vegetables, fruits, herbs, nuts, beans, whole grains, and seafood, but also includes moderate amounts of dairy, meat, and eggs. This plant-based dietary pattern is rich in anti-inflammatory nutrients, fiber, and phytochemicals. Previous studies have shown that a plant-based diet low in animal-based and ultra-processed foods may be helpful to slow the progression of CKD and delay the need for dialysis, via the reduction of cardiometabolic risk factors such as hypertension, CVD, diabetes, and obesity [21,22,23,24,25]. Several randomized controlled clinical trials have confirmed that replacing a dietary saturated fat intake with vegetable polyunsaturated fats, as in the Mediterranean diet, reduces cardiovascular disease incidence by approximately 30%, which is similar to the decrease induced by statins [26,27]. In this study, we have demonstrated that in a high CVD-risk population, such as in those with chronic kidney disease (CKD), these changes will bring alterations in lipid metabolism, particularly in circulating apolipoproteins [28]; in particular, a statistically significant decrease in ApoA-I, ApoA-II, ApoB-100, ApoE, ApoM, and ApoL1 and a statistically significant increase in ApoA-IV, ApoC-III, and ApoH. An imbalance in blood lipid metabolism is thought to contribute to an increased risk of CVD in aCKD patients, which primarily manifests as increases in plasma triglycerides and reductions in high-density lipoprotein (HDL) cholesterol, with little change in low-density lipoprotein (LDL) cholesterol [29]. Nonetheless, atherosclerotic cardiovascular disease (ASCVD) remains in the context of chronic kidney disease (CKD), despite treatment with statins [30]. A meta-analysis of 13 randomized controlled trials of statins in CKD found that the response to statins diminishes in the later stages of CKD [31]. In our study, we observed a lipid imbalance despite more than 50% of patients being treated with statins, which highlights that there is obvious room for a greater reduction in ASCVD risk in CKD beyond the lowering of LDL-C with statins.

The traditional Mediterranean diet contains less than half the amount of choline and L-carnitine molecules when compared to a typical Western diet [32]. The production of tri-methylamine N-oxide (TMAO), a gut microbial metabolite, from dietary choline and L-carnitine has been demonstrated to enhance the likelihood of developing cardiovascular disease in both murine models and humans, operating independently of traditional cardiometabolic risk factors. Carnitine, on the other hand, has a pivotal role in fatty acid β-oxidation and energy production by transporting long-chain fatty acids from the cytoplasm to mitochondria [33]. Both these molecules were also altered in our aCKD population, and these results are in accordance with those previously reported by other researchers [34,35]. A number of carnitine metabolites were identified in the 67-metabolite signature that was proposed for monitoring adherence to the Mediterranean diet [20]. Altogether, these results highlight the potential positive impact that a reduction in the content of choline and L-carnitine in tailored aCKD diets may have on lipid metabolism in this population.

The enrichment analysis performed with metabolomic data on serum samples revealed that linoleic acid metabolism is altered in aCKD patients. Specifically, nine metabolites were reduced in aCKD patients compared to the control group. The use of statins enhances the conversion of linoleic acid to long-chain polyunsaturated fatty acid derivatives [36], which could explain the reduction of linoleic acid and derivates in aCKD patients. However, we performed an analysis according to the use of statins in aCKD patients (Supplementary Figure S6), grouped according to whether they receive statins or not; they showed reduced levels of each metabolite in linoleic acid metabolism compared to the healthy group. Recently, Szczuko et al. demonstrated that the course of inflammation in CKD occurs through a decrease in polyunsaturated fatty acids (PUFA) and the synthesis of monounsaturated fatty acids (MUFA) [37]. In particular, the index C18:3n6/C22:4n6 (gamma linoleic acid/docosatetraenoate) was defined as a new marker in the progression of the disease [37]. Furthermore, dietary conjugated linoleic acid treatment reduces the inflammation observed in chronic kidney disease animal models, due to the inhibition of COX2, and, furthermore, the reduction of prostanoid levels [38,39]. Huang et al. demonstrated, using Swedish dialysis patients, that the proportion of plasma phospholipid linoleic acid was inversely associated with inflammation (IL-6) and all-cause mortality [40], while the low dietary consumption of linoleic acid has been correlated with an increased risk of diabetic kidney disease [41]. Altogether, these results indicate that aCKD patients could benefit from an increased intake of vegetable oils, the primary source of linoleic acid in the Mediterranean diet. The Mediterranean diet is rich in MUFA from olive oil and low in saturated fats from meat and dairy products. The plasma metabolome of patients on the Mediterranean diet is characterized by an increase in unsaturated lipid metabolites [20]. For that reason, the Mediterranean diet is widely recognized for its benefits in reducing cardiovascular risk factors by improving the lipid profile [8].

Endothelial disease and cardiovascular disease progression are largely immune-mediated [42]. It is recognized that CKD is characterized by a remarkable increase in pro-inflammatory cytokine levels, in particular, tumor necrosis factor α (TNFα) and interleukin 6 (IL-6) [43]. Interestingly, CKD has been associated with an increased risk of infection, due to the dysregulation of the innate immune system [44]. In our study, several components from the innate immune system were altered in aCKD patients, including the complement cascade, neutrophil degranulation, Toll-like receptor cascade, and antimicrobial peptide. The uremic milieu alters the ability of endothelial cells to control the alternative complement pathways, which, in turn, amplifies endothelial injuries [45]. These factors are likely to explain the observed imbalances in complement protein expression that were observed in our aCKD population, with some showing an increase while others presented reduced protein expression.

There are compelling data regarding the efficiency of the Mediterranean diet in modulating the gut microbiota. This modulation results in increased microbial diversity and alterations in the proportions of certain bacterial species [46]. Furthermore, a diet comprising a high proportion of fiber-rich foods has also been shown to have a beneficial impact on the integrity of the intestinal barrier [47]. Over the last few years, a group of uremic toxins that are generated in the gut, which potentially connect CKD and the occurrence of CVD, has been described as a risk factor for CKD [48]. The thrombolome is a phenomenon in which CKD-associated dysbiosis and its effect via the generation of gut microbial metabolites induces the prothrombotic phenotype [49]. Our data confirmed the increased abundance of eight well-characterized uremic toxins, including beta-2-microglobulin, prostaglandin-H2 D-isomerase, and cystatin-C [50,51], as well as an upregulation of vWF, in aCKD patients. Other deregulations in the gut microbiota may play a role in vascular and bone disease in CKD [52,53]. These data highlight the relevance of the development of controlled intervention studies on gut microbiota composition and activity, to evaluate the impact of a Mediterranean diet on aCKD patients’ thrombolome.

Oxidative stress and inflammation are features of CKD and drivers of CKD progression, as well as the related cardiovascular and other complications [54,55]. Oxidative stress has been proposed to play a major role in the development of endothelial dysfunction through the production of radical oxygen species (ROS), which activate the intracellular signaling pathways [56]. In particular, glutathione peroxidase catalyzes the reduction of hydrogen peroxide and other organic hydroperoxides into water. Therefore, this enzyme protects cell membrane lipids, proteins, and DNA against oxidative stress. In our study, glutathione peroxidase 3 (GPX3) was significantly decreased in aCKD patients, and this change was positively associated with eGFR [57]. Recently, the activation of the glutathione peroxidase pathway in vitro, by using antioxidant enzyme mimetics (ebselen, glutathione peroxidase mimetic; EUK-134 and EUK-118, both superoxide dismutase mimetics) or N-acetylcysteine reduced not only oxidative stress but also the inflammatory process induced by the uremic milieu on the endothelium [58]. The endothelium, under uremic conditions, exhibits a proinflammatory phenotype with an increased expression of adhesion molecules, such as the above-mentioned vWF and tissue factor, and the production of proinflammatory cytokines, which have been reported as key processes in endothelial activation and damage [59,60]. Antioxidant drugs have been shown to have renoprotective effects in animal studies but have not shown significant effects in clinical trials. The evidence indicates that a diet rich in antioxidants, particularly the Mediterranean diet, protects cells and tissues from oxidation and prevents or delays the development of cardiovascular diseases, thereby reducing mortality risk [61].

The myeloperoxidase (MPO) pathway is a major contributor to oxidative stress in aCKD. The MPO pathway and urea deamination could cause carbamylation, a non-enzymatic reaction during which a carbamoyl moiety is added to proteins, peptides, or amino acids. Carbamylation is involved in the pathogenesis of various diseases, such as atherosclerosis, thrombus formation, infections, autoimmune diseases, and kidney diseases, with homocitrulline being a well-recognized biomarker, and the degree of carbamylation was identified to be an important risk factor for cardiovascular events and mortality in patients with aCKD [62,63,64]. We have also identified homocitrulline levels to be increased in aCKD patients. Nutritional therapy, in particular the Mediterranean dietary pattern, induces a decrease in urea levels that has been associated with the reduction of protein carbamylation in CKD [65].

Amino acid supplementation has been postulated as one of the several strategies to reduce carbamylation in aCKD patients. Considering this phenomenon, we have carefully analyzed protein and amino acid metabolism. From the integrative analysis, we identified that beta-alanine metabolism and the protein digestion and absorption pathways were altered in aCKD patients. Four amino acids and derivates (arginine, beta-alanine, cystine, kynurenine, and proline) were increased in aCKD patients, whereas three (serine, tryptophan, and tyrosine) were reduced. The reduced levels of tryptophan and the accumulation of toxic tryptophan metabolites, especially kynurenine, have been described previously in CKD patients [35,66]. Kynurenine may promote atherosclerosis in aCKD by activating oxidative stress and by leukocyte activation in endothelial and vascular smooth muscle cells [67]. Dahabiyeh et al. have suggested that inhibition of the kynurenine pathway could be a promising target to delay progression from CKD to ESRD, with kynurenine being a potential prognostic biomarker to monitor the progression of CKD [68]. Kynurenine may promote atherosclerosis in ESRD by activating oxidative stress and leukocyte activation in endothelial and vascular smooth muscle cells [59]. In addition, altered tryptophan metabolism may precipitate fatigue in patients with CKD, due to a deficit of melatonin [69] and the mitochondrial dysfunction observed in skeletal muscle under uremic conditions [70,71]. Razquin et al. demonstrated that tryptophan-kynurenine pathway metabolites were associated with high risk of heart failure and atrial fibrillation in a 2 case–control study nested within the PREDIMED trial [71,72]. Moreover, an alteration in the impact of the Mediterranean diet, notably when combined with extra virgin olive oil, was evident in the correlation between kynurenine-related metabolites and heart failure. This indicates that the adverse consequences of these metabolites were confined to the control group [63]. Kynurenine is among the metabolites that constitute the distinctive metabolic profile associated with adherence to the Mediterranean diet. In patients following a Mediterranean diet, the levels of this metabolite were diminished [20].

In our study, homocysteine and cystathionine levels were increased in aCKD patients, which is in line with previous studies [73,74]. Hyperhomocysteinemia undoubtedly has a central role in such a prominent cardiovascular burden, promoting atherosclerosis through increased oxidative stress, impaired endothelial function, and the induction of thrombosis. Homocysteine is the key factor in the pattern of nucleic acid methylations in the genome and epigenetic landscape [75]. It has been reported that the machinery that controls methyl transfer reactions is significantly influenced by the metabolic alterations found in the uremic state, since the uremic toxins themselves have been proven to be related to methyl metabolism and sulfur amino acid metabolism [76].

Frailty and sarcopenia are both linked to a reduction in muscle quantity and quality, due to a catabolic state with elevated muscle protein turnover. Plasma 3-methylhistidine (3-MH) has been defined as a biomarker to display elevated muscle protein turnover and as a biomarker for frailty status [77]. 3-MH is formed in the muscle by the post-translational methylation of histidine residues in actin and myosin. During muscle degradation, 3-MH is released, then is not further metabolized, leaving the body through quantitative excretion in the urine. However, patients with CKD accumulate 3-MH in plasma; aCKD patients showed increased levels of 3-MH. Further analysis should be performed to determine if 3-MH could be useful for identifying frailty in CKD patients.

It is important to acknowledge the limitations inherent in this research. The cross-sectional design limits our ability to assess longitudinal changes or disease progression in patient outcomes. Furthermore, this is not an interventional clinical trial. The sample size was limited, and the aCKD patient characteristics were highly restrictive, excluding patients with other conditions associated with endothelial dysfunction and CVD. It is essential to remember that most patients with CKD have other medical conditions that may influence the results.

## 5. Conclusions

Our findings reveal significant dysregulation of the serum metabolome and proteome in aCKD, particularly related to inflammation, the innate immune system, oxidative stress, uremic toxins, dyslipidemia, and acidosis; most of these pathways are associated with CVD. Previous studies in other cohorts demonstrate that high adherence to MD led to profound changes in the metabolome that are associated with favorable cardiometabolic health. MD-induced changes could reverse altered CVD pathways in patients with aCKD; however, further prospective interventional clinical trials would be beneficial in confirming the potential of the Mediterranean diet in preventing or ameliorating the progression of CVD in aCKD patients.

## Figures and Tables

**Table 1:** Epidemiological characteristics.

General Information		aCKD	Control	*p*-Value
	Age	58.5 ± 14.3	48.8 ± 10.7	0.0245
	Gender (Female; %)	8; 42.1	15; 55.6	0.5499
	Systolic blood pressure	132.3 ± 12.2	109.1 ± 33.8	0.0013
	Diastolic blood pressure	75.5 ± 10.0	67.1 ± 20.4	0.2509
	Heart rate	75.5 ± 12.5	61.9 ± 20.0	0.0107
	Respiratory rate	15.9 ± 1.3	14.9 ± 0.8	0.1812
	Body Mass Index (BMI)	28.6 ± 6.2	25.2 ± 2.6	0.0814
Cardiovascular risk (*n*; %)				
	Dyslipidemia	11; 57.9	0; 0.0	<0.0001
	Hypertension	13; 68.4	0; 0.0	<0.0001
	CKD	19; 100.0	0; 0.0	<0.0001
	Stage G4	10; 52.6	0; 0.0	
	Stage G5	9; 47.4	0; 0.0	
Blood parameters				
	Creatinine mg/dL	3.96 ± 1.13	0.73 ± 0.14	<0.001
	eGFR mL/min/1.73 m^2^	14.9 ± 3.33	88.7 ± 3.77	<0.001
	Na mEq/L	141.5 ± 4.2	140.9 ± 1.7	0.0582
	K mEq/L	4.59 ± 0.46	4.35 ± 0.22	0.1125
	Ca mg/dL	9.23 ± 0.53	9.53 ± 0.50	0.3292
	P mg/dL	4.50 ± 0.55	3.45 ± 0.29	0.0022
	Glucose	98.4 ± 9.58	90.2 ± 9.86	0.0475
	Hb g/L	121.2 ± 12.8	144.5 ± 9.7	<0.001
	C-reactive protein	0.66 ± 0.99	0.098 ± 0.19	0.0423
	Total bilirubin mg/dL	0.40 ± 0.11	0.82 ± 0.31	0.001
	Direct bilirubin mg/dL	0.15 ± 0.05	0.33 ± 0.13	0.0039
	AST U/L	17.6 ± 2.72	21.1 ± 4.32	0.0325
	ALT U/L	17.8 ± 9.62	20.9 ± 9.56	0.5512
	GGT U/L	22.0 ± 1.73	18.9 ± 10.1	0.2854
	ALP U/L	120.2 ± 23.4	65.8 ± 16.6	0.0035
	Total Cholesterol	177.8 ± 28.2	190.0 ± 31.4	0.1884
	HDL	44.2 ± 9.63	53.5 ± 16.8	0.0566
	LDL	109.5 ± 17.7	122.3 ± 29.2	0.1136
	Triglycerides	136.6 ± 38.6	108.9 ± 48.4	0.0388

aCKD, Advanced chronic kidney disease; eGFR, estimated glomerular filtrate rate; AST, aspartate aminotransferase; ALT, alanine aminotransferase; GGT, gamma glutamyl transferase; ALP, alkaline phosphatase. Fisher’s exact test and the Mann–Whitney test were performed. *p* < 0.05 indicates significantly different results when compared to the control group.

**Table 2:** The top metabolite biomarkers that are differentially expressed.

	Name	FC	ROC Analysis	
			AUC	*p*-Value
1	Homocitrulline	5.88	1.000	4.12 × 10^−27^
2	1-Methylhistidine	7.54	1.000	6.12 × 10^−24^
3	Cystathione	11.59	0.994	5.57 × 10^−15^
4	Acetyl-carnitine	5.86	0.996	2.33 × 10^−14^
5	Sucrose	18.56	0.979	7.73 × 10^−14^
6	BAIBA	6.40	0.998	1.48 × 10^−13^
7	Kynurenine	2.55	0.979	1.90 × 10^−13^
8	AIBA	2.32	0.984	2.11 × 10^−13^
9	Cortisone	−1.96	0.979	5.00 × 10^−13^
10	2-methyl-butyryl-carnitine/Isovaleryl-carnitine/Valeryl-carnitine	2.61	0.988	8.59 × 10^−12^
11	Homocystine	6.10	0.969	6.79 × 10^−10^
12	3-Methylhistidine	5.85	0.899	1.15 × 10^−8^
13	Methylglutaryl-carnitine/Adipoyl-carnitine	6.81	0.922	1.64 × 10^−8^
14	EpOME-iso3	−3.57	0.922	2.46 × 10^−8^
15	Octenoyl-carnitine	3.06	0.903	1.23 × 10^−7^
16	Androstenedione	3.10	0.832	4.53 × 10^−5^
17	LPI 14:0	−4.07	0.793	2.54 × 10^−4^
18	LPC 18:3-sn2	−3.42	0.782	2.66 × 10^−4^
19	LPC 18:3-sn1	−3.04	0.774	3.76 × 10^−4^
20	12-HETE	−3.48	0.743	2.83 × 10^−3^

**Table 3:** Altered metabolites from linoleic acid metabolism in aCKD patients. Lecithin (phosphatidylcholine), Linoleic acid, 9(10)EpOME, 12(13)EpOME, 9(10)-DiHOME, 12(13)-DiHOME, 9-oxoODE, 9-HODE/13-HODE, and dihomo-γ-linoleic.

Name	Control	aCKD	diff LOG2	FC	q-Value (FDR)
EpOME-iso3	−6.736 (0.865)	−8.572 (0.960)	0.280	−3.570	0.000
EpOME-iso2	−7.156 (0.445)	−7.644 (0.531)	0.713	−1.402	0.010
9(10)-DiHOME	−7.045 (0.810)	−8.020 (0.704)	0.509	−1.966	0.001
Linoleic acid-iso1	6.462 (0.627)	5.767 (0.735)	0.618	−1.619	0.008
9-HODE/13-HODE	−5.588 (1.068)	−6.238 (0.497)	0.637	−1.569	0.012
9-OxoODE	−6.650 (0.843)	−7.291 (0.609)	0.641	−1.559	0.003
12(13)-DiHOME	−6.814 (0.550)	−7.457 (0.653)	0.640	−1.562	0.006
N-Linoleoyl ethanolamide	3.658 (0.469)	3.250 (0.474)	0.754	−1.327	0.021
dihomo-gamma-linolenic acid	−0.076 (0.415)	−0.409 (0.410)	0.794	−1.260	0.032
Lecithin (PhosphatidylCholine)					
PC 34:2	7.836 (0.271)	7.609 (0.227)	0.854	−1.170	0.018
PC 36:2	7.021 (0.316)	6.743 (0.323)	0.825	−1.213	0.020
PC 31:0	−2.186 (0.540)	−2.648 (0.525)	0.726	−1.377	0.021
PC 38:3 e	−2.770 (0.601)	−3.265 (0.463)	0.710	−1.409	0.017
PC 36:2 e	−1.158 (0.437)	−1.656 (0.451)	0.708	−1.412	0.004
PC 30:0	−0.397 (0.769)	−1.134 (0.637)	0.600	−1.667	0.016
PC 32:2	0.144 (0.799)	−0.714 (0.686)	0.552	−1.813	0.014

**Table 4:** The top protein biomarkers that are differentially expressed.

Swiss-Prot ID	Name	FC	ROC Analysis	
			AUC	*p*-Value
P61769	Beta-2-microglobulin (B2M)	3.104	1	8.41 × 10^−21^
P01034	Cystatin-C (CST3)	2.433	1	1.71 × 10^−20^
P22692	Insulin-like growth factor-binding protein 4 (IGFBP4)	1.985	1	1.31 × 10^−18^
P22352	Glutathione peroxidase 3 (GPX3)	−1.889	1	4.33 × 10^−17^
P06727	Apolipoprotein A-IV (ApoA4)	2.390	1	1.95 × 10^−17^
P36955	Pigment epithelium-derived factor	1.690	1	9.57 × 10^−16^
P61626	Lysozyme C (LYZ)	3.061	0.998	9.41 × 10^−16^
P00746	Complement factor D (CFD)	2.145	0.996	1.58 × 10^−16^
P41222	Prostaglandin-H2 D-isomerase (β-trace protein)	2.882	0.981	3.26 × 10^−14^
P16070	CD44 antigen	1.519	0.981	8.24 × 10^−11^
Q6EMK4	Vasorin	1.409	0.959	4.27 × 10^−11^
P02753	Retinol-binding protein 4 (RBP4)	1.876	0.975	1.03 × 10^−11^
P02760	Protein AMBP (AMBP)	1.331	0.963	1.58 × 10^−10^
P02749	Beta-2-glycoprotein 1 (ApoH)	1.449	0.986	1.27 × 10^−10^
Q6UXB8	Peptidase inhibitor 16 (PI16)	1.491	0.945	5.99 × 10^−9^
P43251	Biotinidase	−1.302	0.926	4.61 × 10^−8^
P08697	Alpha-2-antiplasmin	−1.244	0.924	4.09 × 10^−8^
P02652	Apolipoprotein A-II (ApoA2)	−1.320	0.932	3.56 × 10^−8^
P05160	Coagulation factor XIII B (F13B)	1.235	0.930	1.75 × 10^−8^
P02751	Fibronectin (FN1)	−1.504	0.889	6.91 × 10^−7^

**Table 5:** Proteins from the complement and coagulation cascades that are differentially expressed in aCKD patients compared to the control group.

Name	Control	aCKD	diff LOG2	FC	q-Value (FDR)
Complement cascade					
Complement factor D	−0.288 (0.230)	0.813 (0.351)	1.101	2.15	0.000
Complement factor H-related protein 2	−0.218 (0.763)	0.471 (0.611)	0.689	1.61	0.000
Complement C4-B	0.024 (0.368)	0.477 (0.537)	0.453	1.37	0.005
C4b-binding protein alpha chain	0.021 (0.512)	0.474 (0.777)	0.453	1.37	0.028
Complement component C9	−0.132 (0.267)	0.308 (0.329)	0.44	1.36	0.000
Coagulation factor XIII B chain	0.089 (0.141)	0.394 (0.158)	0.305	1.24	0.000
von Willebrand factor	−0.682 (0.320)	−0.433 (0.392)	0.249	1.19	0.049
Coagulation factor XI	0.152 (0.333)	0.369 (0.234)	0.217	1.16	0.041
Vitamin K-dependent protein S	0.136 (0.275)	0.315 (0.200)	0.179	1.13	0.033
Coagulation factor V	0.077 (0.229)	0.236 (0.166)	0.159	1.12	0.031
Coagulation factor IX	0.039 (0.161)	0.185 (0.174)	0.146	1.11	0.013
Plasma protease C1 inhibitor (SERPING1)	0.184 (0.138)	0.329 (0.156)	0.145	1.11	0.006
Kininogen-1	0.025 (0.159)	0.135 (0.124)	0.11	1.08	0.036
Coagulation cascade					
Carboxypeptidase B2	0.133 (0.174)	−0.021 (0.224)	−0.154	−1.11	0.029
Complement component C8 gamma chain	0.110 (0.204)	−0.043 (0.204)	−0.153	−1.11	0.038
Plasminogen	0.140 (0.155)	−0.029 (0.129)	−0.169	−1.12	0.001
Prothrombin	0.188 (0.131)	0.022 (0.140)	−0.166	−1.12	0.001
Complement C3	0.090 (0.150)	−0.072 (0.219)	−0.162	−1.12	0.012
Coagulation factor XII	0.082 (0.339)	−0.185 (0.334)	−0.267	−1.20	0.011
Vitronectin	−0.069 (0.258)	−0.364 (0.157)	−0.295	−1.23	0.000
Alpha-2-antiplasmin (SERPINF2)	0.205 (0.164)	−0.110 (0.150)	−0.315	−1.24	0.000
Fibronectin	0.008 (0.365)	−0.581 (0.298)	−0.589	−1.50	0.000

**Table 6:** Proteins from the innate immune system that are differentially expressed in aCKD patients compared to the control group. Several pathways from the innate immune system contain these proteins: the complement cascade, neutrophil degranulation, antimicrobial peptide, and Toll-like receptor cascade.

Name	Control	aCKD	diff LOG2	FC	q-Value (FDR)
Complement cascade					
Carboxypeptidase N catalytic chain	0.104 (0.159)	−0.142 (0.193)	−0.246	−1.19	0.000
Carboxypeptidase N subunit 2	0.138 (0.178)	−0.108 (0.211)	−0.246	−1.19	0.001
Complement C3	0.090 (0.150)	−0.072 (0.219)	−0.162	−1.12	0.012
Complement C4-B	0.024 (0.368)	0.477 (0.537)	0.453	1.37	0.005
C4b-binding protein alpha chain	0.021 (0.512)	0.474 (0.777)	0.453	1.37	0.028
Complement component C8 gamma chain	0.110 (0.204)	−0.043 (0.204)	−0.153	−1.11	0.038
Complement component C9	−0.132 (0.267)	0.308 (0.329)	0.44	1.36	0.000
Carboxypeptidase B2	0.133 (0.174)	−0.021 (0.224)	−0.154	−1.11	0.029
Plasma protease C1 inhibitor (SERPING1)	0.184 (0.138)	0.329 (0.156)	0.145	1.11	0.006
Vitronectin	−0.069 (0.258)	−0.364 (0.157)	−0.295	−1.23	0.000
Complement factor D	−0.288 (0.230)	0.813 (0.351)	1.101	2.15	0.000
Prothrombin	0.188 (0.131)	0.022 (0.140)	−0.166	−1.12	0.001
Neutrophil degranulation					
Alpha-1-acid glycoprotein 1	−0.101 (0.372)	0.520 (0.460)	0.621	1.54	0.000
Alpha-1-acid glycoprotein 2	0.080 (0.334)	0.449 (0.248)	0.369	1.29	0.001
Leucine-rich alpha-2-glycoprotein	−0.276 (0.369)	0.333 (0.412)	0.609	1.53	0.000
Cystatin-C	−0.287 (0.258)	0.996 (0.261)	1.283	2.43	0.000
Complement factor D	−0.288 (0.230)	0.813 (0.351)	1.101	2.15	0.000
Lysozyme C	−0.443 (0.407)	1.171 (0.485)	1.614	3.06	0.000
CD44 antigen	−0.164 (0.232)	0.439 (0.246)	0.603	1.52	0.000
Gelsolin	0.119 (0.149)	0.378 (0.250)	0.259	1.20	0.001
Lipopolysaccharide-binding protein	−0.180 (0.435)	0.201 (0.340)	0.381	1.30	0.008
Alpha-1B-glycoprotein	−0.074 (0.127)	−0.225 (0.135)	−0.151	−1.11	0.001
Beta-2-microglobulin	−0.490 (0.299)	1.144 (0.357)	1.634	3.10	0.000
Antimicrobial peptide					
N-acetylmuramoyl-L-alanine amidase	0.135 (0.244)	−0.037 (0.217)	−0.172	−1.13	0.017
Lysozyme C	−0.443 (0.407)	1.171 (0.485)	1.614	3.06	0.000
Toll-like receptor cascade					
Monocyte differentiation antigen CD14	−0.048 (0.244)	0.239 (0.282)	0.287	1.22	0.002
Lipopolysaccharide-binding protein	−0.180 (0.435)	0.201 (0.340)	0.381	1.30	0.008

**Table 7:** Proteins from cholesterol metabolism that are differentially expressed in aCKD patients compared to the control group. Furthermore, the integrative analysis showed 7 additional altered metabolites: glycocholic acid iso-4, TG52:1, TG52:2, TG52:3, TG54:2, TG54:3, and TG54:4.

Name	Control	aCKD	diff LOG2	FC	q-Value (FDR)
Proteins					
Apolipoprotein A-IV (APOA4)	−0.216 (0.249)	1.041 (0.375)	1.257	2.39	0.000
Beta-2-glycoprotein 1 (APOH)	0.058 (0.248)	0.593 (0.151)	0.535	1.45	0.000
Apolipoprotein C-III (APOC3)	−0.231 (0.380)	0.161 (0.407)	0.392	1.31	0.005
Apolipoprotein M	0.259 (0.280)	−0.246 (0.284)	−0.505	−1.42	0.000
Apolipoprotein L1	−0.158 (0.386)	−0.756 (0.400)	−0.598	−1.51	0.000
Apolipoprotein A-II (APOA2)	0.258 (0.200)	−0.142 (0.201)	−0.4	−1.32	0.000
Apolipoprotein E (APOE)	−0.054 (0.348)	−0.382 (0.425)	−0.328	−1.26	0.016
Apolipoprotein A-I (APOA1)	0.098 (0.168)	−0.150 (0.255)	−0.248	−1.19	0.001
Apolipoprotein B-100 (APOB)	0.033 (0.295)	−0.200 (0.273)	−0.233	−1.18	0.024
Metabolites					
TG54:3	7.419 (0.704)	8.258 (0.994)	1.789	1.789	0.010
TG52:1	4.279 (0.886)	5.090 (1.253)	1.754	1.754	0.039
TG54:2	4.805 (0.785)	5.484 (1.030)	1.601	1.601	0.042
TG54:4	7.961 (0.691)	8.621 (0.623)	1.580	1.580	0.010
TG52:2	9.512 (0.648)	10.124 (0.831)	1.528	1.528	0.024
TG52:3	9.487 (0.664)	10.012 (0.546)	1.439	1.439	0.023
Glycocholic acid-iso4	−0.708 (1.236)	−2.079 (1.589)	0.387	−2.586	0.011

**Table 8:** Integrative pathway analysis.

	Total	Hits	Proteins	Metabolites	Expected	Raw p	−log(p)	Holm Adjust	FDR	Impact
Complement and coagulation cascades	80	21	21	0	0.656	9.5297 × 10^−27^	59.915	3.1543 × 10^−24^	3.1543 × 10^−24^	0.731
Linoleic acid metabolism	57	9	0	9	0.467	7.3207 × 10^−10^	21.035	2.4158 × 10^−7^	1.2116 × 10^−7^	0.683
Cholesterol metabolism	60	9	7	2	0.492	1.179 × 10^−9^	20.559	3.879 × 10^−7^	1.3009 × 10^−7^	0.075
African trypanosomiasis	45	6	3	3	0.369	1.621 × 10^−6^	13.332	0.00053169	0.00013414	0.028
Fat digestion and absorption	54	6	3	3	0.442	4.8446 × 10^−6^	12.238	0.0015842	0.00032071	0
Protein digestion and absorption	142	8	1	7	1.160	2.0511 × 10^−5^	10.795	0.0066865	0.0011315	0
Vitamin digestion and absorption	63	5	4	1	0.516	0.00016024	8.7388	0.052079	0.0075772	0
Amoebiasis	115	6	4	2	0.942	0.00035372	7.947	0.11461	0.014635	0.017
Biosynthesis of unsaturated fatty acids	79	5	0	5	0.647	0.00046354	7.6766	0.14972	0.017048	0.030
PPAR signaling pathway	81	5	4	1	0.664	0.00052024	7.5612	0.16752	0.01722	0.153
Pertussis	86	5	5	0	0.705	0.00068479	7.2864	0.21982	0.020606	0.176
Staphylococcus aureus infection	98	5	5	0	0.804	0.0012364	6.6956	0.39564	0.034104	0.442
beta-Alanine metabolism	63	4	1	3	0.516	0.0017406	6.3535	0.55527	0.041442	0.759
Central carbon metabolism in cancer	106	5	0	5	0.869	0.0017528	6.3465	0.5574	0.041442	0

## Data Availability

All relevant materials are presented in the present manuscript.

## Supplementary Materials

The following supporting information can be downloaded at: https://www.mdpi.com/article/10.3390/nu16213739/s1, The metabolomics and proteomics quantification data reported in this study are available as Supplementary Materials: Supplementary Methods, Supplementary Figures and Supplementary Tables. Figure S1. Supervised analysis based on metabolomics datasets; Figure S2. Unsupervised analysis from protein datasets; Figure S3. Supervised analysis from proteomics datasets; Figure S4. Altered elements from the innate immune system (Reactome–HAS-168249) in aCKD patients; Figure S5. Altered proteins from cholesterol metabolism (KEGG-hsa04979) in aCKD patients; Figure S6. Impact of statins on metabolites from linoleic acid metabolism. Table S1. List of metabolites quantified; Table S2. List of the proteins identified; Table S3. Altered metabolites in aCKD patients compared to the control group; Table S4. PLS-DA and OPLS-DA data for each metabolite; Table S5. Enrichment analysis using metabolomic data; Table S6. Altered proteins in aCKD patients compared to the control group; Table S7. Uremic toxins identified in our study; Table S8. PLS-DA and OPLS-DA data for each protein; Table S9. Enrichment analysis using proteomic data; Table S10. GO—Biological process; Table S11. GO—Cellular component; Table S12. GO—Molecular function; Table S13. Complete integrative analysis; Table S14. List of the pure chemical standards used to generate internal standard calibration curves in the metabolomic analysis.
