# Supplementary material for: Mediterranean Diet Pattern: Potential Impact on the Different Altered Pathways Related to Cardiovascular Risk in Advanced Chronic Kidney Disease
Source: Nutrients. 2024 Oct 31;16(21):3739. doi: 10.3390/nu16213739 (PMC11547550; doi:10.3390/nu16213739)
Supplement: Supplementary file 1 [file nutrients-16-03739-s001.zip › nutrients-3253014-Supplementary_Methods_Figures.pdf]

Supplementary Materials

SUPPLEMENTARY METHODS

Metabolomic Analysis

Method 1: Lipidomic Analysis with Methanol Extraction

For the extraction of less hydrophobic lipids, a protein precipitation extraction was performed by adding four volumes of methanol containing internal standard mixture (myristic acid-d27, arachidonic acid-d8, Cholic acid-d5, Taurocholic acid-d5 LysoPC 18:1-d7, and labelled Carnitine Mix from Cambridge Isotopes) to serum. Then, the samples were mixed and incubated at -20ºC for 30 min, centrifuged at 15,000 rpm and supernatant was analysed by UHPLC-qTOF (model 6550 of Agilent, USA) in both positive and negative electrospray ionization mode.

The chromatographic method was the same for both ionization modes. A gradient elution with water and acetonitrile with 0.05% formic acid as mobile phase and C18 column (ACQUITY UPLC BEH C18 Column, 1.7µm, 2.1mmX100mm) that allowed the sequential elution of the less hydrophobic lipids such as non-esterified fatty acids, acyl carnitines, bile acids, steroids and lysophospholipids among others.

The identification of lipid species was performed by matching their accurate mass and tandem mass spectrum, when available, to Metlin-PCDL from Agilent containing more than 40,000 metabolites and lipids. In addition, chromatographic behavior of pure standards for each family and bibliographic information was used to ensure their putative identification.

After putative identification of lipids, these were semiquantified depending on their family similarity by an internal standard calibration curves using the pure chemical standards indicated in the Table S14A.

Method 2: Lipidomic Analysis with Choloform: Methanol (Folch Method) Extraction

For the extraction of more hydrophobic lipids, a liquid-liquid extraction with chloroform:methanol (2:1) based on Folch procedure was performed by adding four volumes of chloroform:methanol (2:1) containing internal standard mixture (Lipidomic SPLASH®) to serum. Then, the samples were mixed and incubated at -20ºC for 30 min. Afterwards, water with NaCl (0.8 %) was added and mixture was centrifuged at 15,000 rpm. Lower phase was recovered, evaporated to dryness and reconstituted with methanol:methyl-tert-butyl ether (9:1) and analysed by UHPLC-qTOF (model 6550 of Agilent, USA) in both positive and negative electrospray ionization modes.

As method 1, the chromatographic method was the same for both ionizations. The gradient consists in an elution with a ternary mobile phase containing water, methanol and 2-propanol with 10mM ammonium formate and 0.1% formic acid. The stationary phase was a C18 column (Kinetex EVO C18 Column, 2.6 µm, 2.1 mm X 100 mm) that allows the sequential elution of the more hydrophobic lipids such as lysophospholipids, sphingomyelins, phospholipids, diglycerides, triglycerides, and cholesteryl esters, among others.

The identification of lipid species was performed by matching their accurate mass and tandem mass spectrum, when available, to Metlin-PCDL from Agilent containing more than 40,000 metabolites and lipids. In addition, chromatographic behaviour of pure standards for each family and bibliographic information was used to ensure their putative identification.

After putative identification of lipids, these were semiquantified depending on their family similarity by and internal standard calibration curves using the pure chemical standards indicated in the Table S14B.

Method 3: Aminoacids Analysis

For the extraction of aminoacids, a protein precipitation extraction was performed by adding four volumes of methanol containing internal standards (Metabolomics labelled aminoacid mixture from Cambridge Isotopes) to serum samples. Then, the samples were mixed and incubated at -20ºC for 30min, centrifuged at 15,000rpm and supernatant was derivatized using AccQ-Tag reagent from Waters® following manufacturing protocol. Then, derivatized aminoacids were analysed by UHPLC-QqQ (model 6490 of Agilent, USA) in Multiple Reaction Monitoring acquisition.

The chromatographic separation consists of a gradient elution with water and acetonitrile with 0.1% formic acid as mobile phase and C18 column (ACQUITY UPLC HSS T3 Column, 1.7µm, 2.1mmX150mm) that allows the determination and separation of aminoacids and derivatives. Their semi-quantification was done with pure chemical standard curves for all compounds.

Method 4: Polar Metabolites in Central Carbon Metabolism

For the extraction of polar metabolites in central carbon metabolism, a protein precipitation extraction was performed by adding eight volumes of methanol:water (8:2) containing internal standard mixture (succinic acid-d4, myristic acid-d27, glicerol-13C3 and D-glucose-13C6) to serum samples. Then, the samples were mixed and incubated at 4ºC for 10min, centrifuged at 15,000rpm and supernatant was evaporated to dryness and freeze dried in a liophylizator before compound derivatization (metoximation and silylation). The derivatized compounds were analysed by GC-qTOF (model 7200 of Agilent, USA).

The chromatographic separation was based on Fiehn Method, using a J&W Scientific HP5-MS (30m x 0.25mm i.d., 0.25µm film capillary column and helium as carrier gas using an oven program from 60 to 325ºC. Ionization was done by electronic impact (EI), with electron energy of 70eV and operated in full Scan mode.

In addition to targeted compounds from central carbon metabolism, a screening for the identification of more metabolites was performed by matching their EI mass spectrum and retention time to metabolomic Fiehn library (from Agilent) which contains more than 1,400 metabolites. After putative identification of metabolites, these and target compounds were semi-quantified depending on their family similarity by an internal standard calibration curves using the pure chemical standards indicated in the Table S14C.

After metabolomic analyses, lipidomic method based on methanol extraction provided semi-quantitative results of 204 lipids, lipidomic method based on Folch extraction provided 119 lipids, aminoacid method provided semi-quantitative results of 49 aminoacids and derivatives, and polar metabolites in central carbon metabolism method provided 28 additionally compounds. Thus, 400 unique metabolites were successfully quantified in the serum samples analyzed and can be found in the Table S1. The most common name is indicated except for the case of some lipids were abbreviated name is used as follows: LPC for lysophosphocholine (n=43), LPE for lysophosphoethanolamine (n=35), LPI for lysophoshoinositole (n=12), ChoE for cholesteryl esters (n=15), DG for diglyceride (n=7), TG for triglyceride (n=25), SM for sphingomyelin (n=23), and PC for phosphatidylcholine (n=45). The first number indicates the acyl carbon atoms, and the second indicates the number of insaturations. Some compounds can be determined in more than one analysis, and then the most confident results were used.

Proteomic Analysis

Protein Extraction and Quantification

Before the proteomic analysis, the seven most abundant serum proteins (Albumin, IgG, antitrypsin, IgA, transferrin, haptoglobin and fibrinogen) were depleted to increase the number of identified/quantified proteins. Thus, 12µl of each sample were passed twice through the Human-7 Multiple Affinity Removal Spin (MARS) cartridge from Agilent Technologies and the flow through fractions were collected for proteomic analysis following manufacturer protocol. Flow through fractions were concentrated and buffer

exchanged to about 10µl of 6M urea in 50mM ammonium bicarbonate (ABC) by using 5K MWCO spin columns (Agilent 5185-5991).

Protein Digestion and Peptide 10-Plex TMT Labeling

30µg of total protein were reduced with 4mM 1.4-Dithiothreitol (DTT) for 1h at 37°C and alkylated adding 8mM iodoacetamide (IAA) for 30min at 25ºC in the dark. Afterwards, samples were overnight digested (pH 8.0, 37ºC) with sequencing-grade trypsin (Promega) at enzyme:protein ratio of 1:50. Digestion was quenched by acidification with 1% (v/v) formic acid and peptides were desalted on Oasis HLB SPE column (Waters) before TMT 10-plex labelling (Thermo Fisher) following manufacturer instructions.

To normalize all samples in the study along the different TMT-multiplexed batches used, a pool containing all the samples was labelled with TMT-pool Tag and included in each TMT batch. The different TMT 10-plex batches were desalted on Oasis HLB SPE columns before the nanoLC-MS analysis.

NanoSCX-nanoLC-(Orbitrap)MS/MS Analysis

Labelled and multiplexed samples were on-line fractionated in a strong cation exchange (SCX) nano-column (Agilent) by gradient salt pulsed sequential elution using ammonium acetate (NH4AcO). Thus, 6 fractions were analyzed for each TMT-plex using 0, 12.5, 25, 50, 100 and 500mM NH_4AcO. Each SCX fraction eluted were desalted on a trap nano-column (100µm I.D.; 2cm length; 5µm particle diameter, Thermo Fisher Scientific, San José, CA, USA) and separated onto a C-18 reversed phase (RP) nano-column (75µm I.D.; 15cm length; 3µm particle diameter, Nikkyo Technos Co. LTD, Japan) on an EASY-II nanoLC from Thermo Fisher. The chromatographic separation was performed with a 90min gradient using Milli-Q water (0.1% formic acid) and acetonitrile (0.1% formic acid) as mobile phase at a flow rate of 300nL/min.

Mass spectrometry analyses were performed on an LTQ-Orbitrap Velos Pro from Thermo Fisher by an enhanced FT-resolution MS spectrum (R=30,000 FHMW) followed by a data dependent FT-MS/MS acquisition (R=15,000 FHMW, 40% HCD) from the most intense ten parent ions with a charge state rejection of one and dynamic exclusion of 0.5min.

Protein Identification/Quantification

Protein identification/quantification was performed on Proteome Discoverer software v.1.4.0.288 (Thermo Fisher) by Multidimensional Protein Identification Technology (MudPIT) combining the 6 raw data files obtained after SCX fractionation. For protein identification, all MS and MS/MS spectra were analyzed using Mascot search engine (v.2.5). Mascot was set up to search SwissProt_2018_03 FASTA database (557012 entries), restricting for Human taxonomy (20317 sequences) and assuming trypsin digestion. Two missed cleavages were allowed and an error of 0.02Da for FT-MS/MS fragmentation mass and 10.0ppm for a FT-MS parent ion mass were allowed. TMT-10plex was set as quantification modification and oxidation of methionine and acetylation of N-termini were set as dynamic modifications, whereas carbamidomethylation of cysteine was set as static modifications. The false discovery rate (FDR) and protein probabilities were calculated by Perclorator.

For protein quantification, the ratios between each TMT-label against TMT-Pool label were used and quantification results were normalized based on protein median.

After proteomic analysis, a total of 273 proteins were identified on the samples analyzed. These proteins and information regarding relative quantification and identification such as Mascot score, protein coverage and identified unique and total peptides are shown in the Table S2.

Statistical Analysis

Data Pre-Processing

On initial metabolomic and proteomic analysis, readers were blinded to patient's status. For statistical analyses only those proteins and metabolites that were present in ≥70% of the samples in at least one group were considered. In addition, a log base 2 transformation was applied to the protein data. Finally, data were mean-centered, and Pareto scaled.

Multivariate Statistical Analysis

Initially, a multivariate statistical approach was performed using Metaboanalyst 4.0 (http://www.metaboanalyst.ca/). The modelling included the use of unsupervised methods such a principal component analysis (PCA) and hierarchical clustering (HCA), and supervised methods, that included partial least squares discriminant analysis (PLS-DA) and an orthogonal projection to latent structures discriminant analysis (OPLS-DA). All these methods were applied with a Pareto scaling. Multivariate techniques are based on the eigen-descomposition of a cross-product matrix (e.g., covariance matrix) and thus require complete datasets. However, in multivariate data it is usually the case that missing values are present. Therefore, they need to be estimated prior to applying any multivariate analysis. Between the several approaches to estimate missing values, we used a Bayesian principal component analysis (BPCA), which works well for values missing at random. It estimates missing values using the observed values without missing values. The PCA is calculated using Bayes theorem and the Bayesian estimation is used to calculate the likelihood of an estimated value.

Unsupervised methods were initially applied to identify trends, groupings, and outliers. Supervised methods incorporate additional information about the samples into the models to identify variation in the data that is correlated with the phenotypic response variables. Biomarkers should have a high reliability. However, peaks with a high reliability but low magnitude/intensity are close to the noise level and there is high risk for spurious correlations. Therefore, ideal biomarkers have high magnitude and high reliability and can be easily identified by both extremes of the S-plot.

Univariate Statistical Analysis

For each protein or metabolite, univariate test was performed. For the univariate case, data were not Pareto scaled. Initially, the Control and ESRD samples were compared. A Kolmogorov-Smirnov test was carried out for each protein to check for distribution normality. Afterwards, either a t-test or a Wilcoxon test was performed depending on each protein's distribution. In the case of a t-test, a test for equality of variances was performed prior to the analysis. The Benjamini-Hochberg method was used to adjust p values for multiple testing with consideration of 5% false discovery rate (FDR). The reported results included the means and standard deviations (SD) for each group, the fold change (FC), and the p and q (p corrected for FDR) values. In addition, a ROC analysis was performed for each protein, and the area under the curve (AUC) and p values are reported.

Pathway Analysis

The metabolites differentially presented in serum samples from ESRD patients were used to identify differential pathways due to an over-representation analysis (ORA) performed in Metaboanalyst using the Metabolite Set Enrichement Analysis (MSEA).

Differential pathways were identified using a method based on protein-protein interaction networks and enrichment analysis. Significantly different proteins based on the univariate analysis were used for the pathway analyses. The pathway networks were identified by exploring the interactions of pathway-enriched genes with the global human protein-protein interaction (PPI) network from the Search Tool for the Retrieval of Interacting Genes/proteins (STRING) database (https://string-db.org/). We constructed

an extended network based on a high confidence score of 0.7. Network nodes represent proteins, whereas edges represent protein-protein associations. The thickness of the edge indicates the confidence, with a higher thickness indicating higher confidence. Proteins were clustered using a Markov Cluster Algorithm (MCL) clustering with an inflation parameter of 1.4. Solid and dotted lines indicate connection within the same and different cluster, respectively. In addition, an enrichment analysis was performed in each displayed network in STRING, testing a number of functional annotation spaces including Gene Ontology (with three categories: Biological Process (GO-BP), Cellular Component (GO-CC), Molecular Function (GO-MF)), Kyoto Encyclopedia of Genes and Genome (KEGG), Reactome, PFAM Protein Domains and InterPro Protein Domains and Features. The results of the enrichment are sorted according by their enrichment P-value, which are corrected for multiple testing using the Benjamini-Hochberg method.

The integrated pathway analysis has been performed with the module Join Pathway Analysis from Metaboanalyst, combining metabolomics and gene expression (proteomics) studies conducted under the same experimental conditions. ORA based on hypergenometrics analysis has been chosen. The topology analysis evaluates whether a given protein or metabolite plays an important role in a biological response based on its position within a pathway. Degree Centrality has been used to measure the number of links that connect to a node (representing either a protein or metabolite) within a pathway.

SUPPLEMENTARY RESULTS

Metabolomic Unsupervised Analysis

The PCA analysis showed a separation between the Control and almost all the aCKD samples. The first principal component explained 35% of the variance in the data, whereas the addition of a second component increased explained variance to almost 45%. These results were corroborated after an HCA analysis, where two independent clusters corresponding the Control and aCKD groups could be identified.

Proteomic Unsupervised Analysis

The PCA analysis showed a clear separation between the Control and aCKD groups without the presence of any outlier (Figure S2A). The first principal component explained 20% of the variance in the data, whereas the addition of a second component increases to explained variance to almost 30%. These results were confirmed in the HCA analysis, where two clear clusters corresponding the Control and aCKD groups could be identified (Figure S2B and S2C).

SUPPLEMENTARY FIGURES

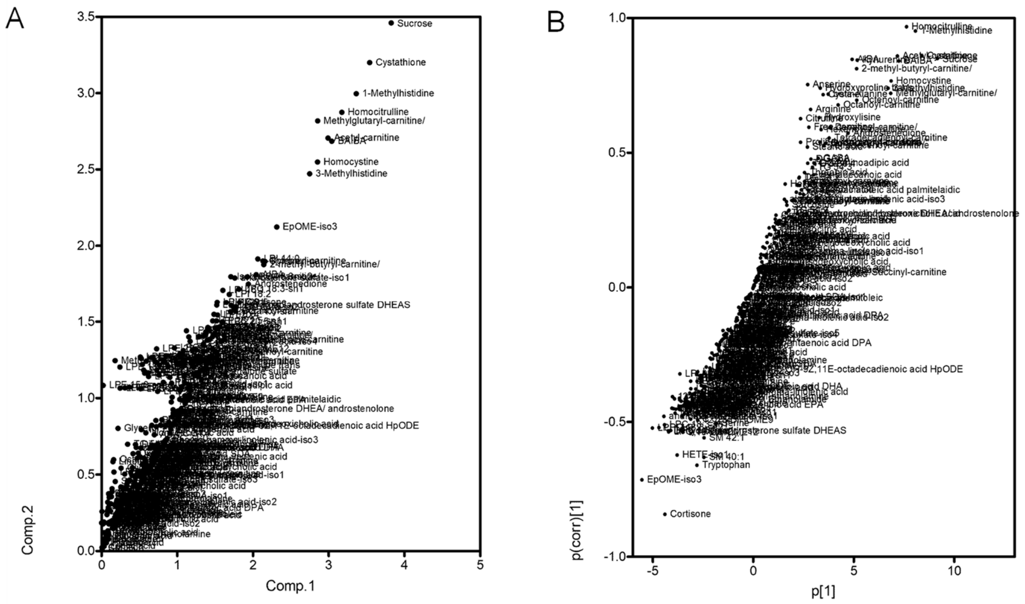

Figure S1. Supervised analysis from metabolomics datasets. (**A**) PLS-DA loading plot for the aCKD group showing the relative contribution of each component to class separation. (**B**) OPLS-DA loading S-plot for the aCKD group showing the relative contribution of each variable to class separation. Each dot corresponds to one protein. The p(corr)[1] axis represents the correlation of the variable towards class separation, whereas the p[1] axis represents the magnitude of the variable.

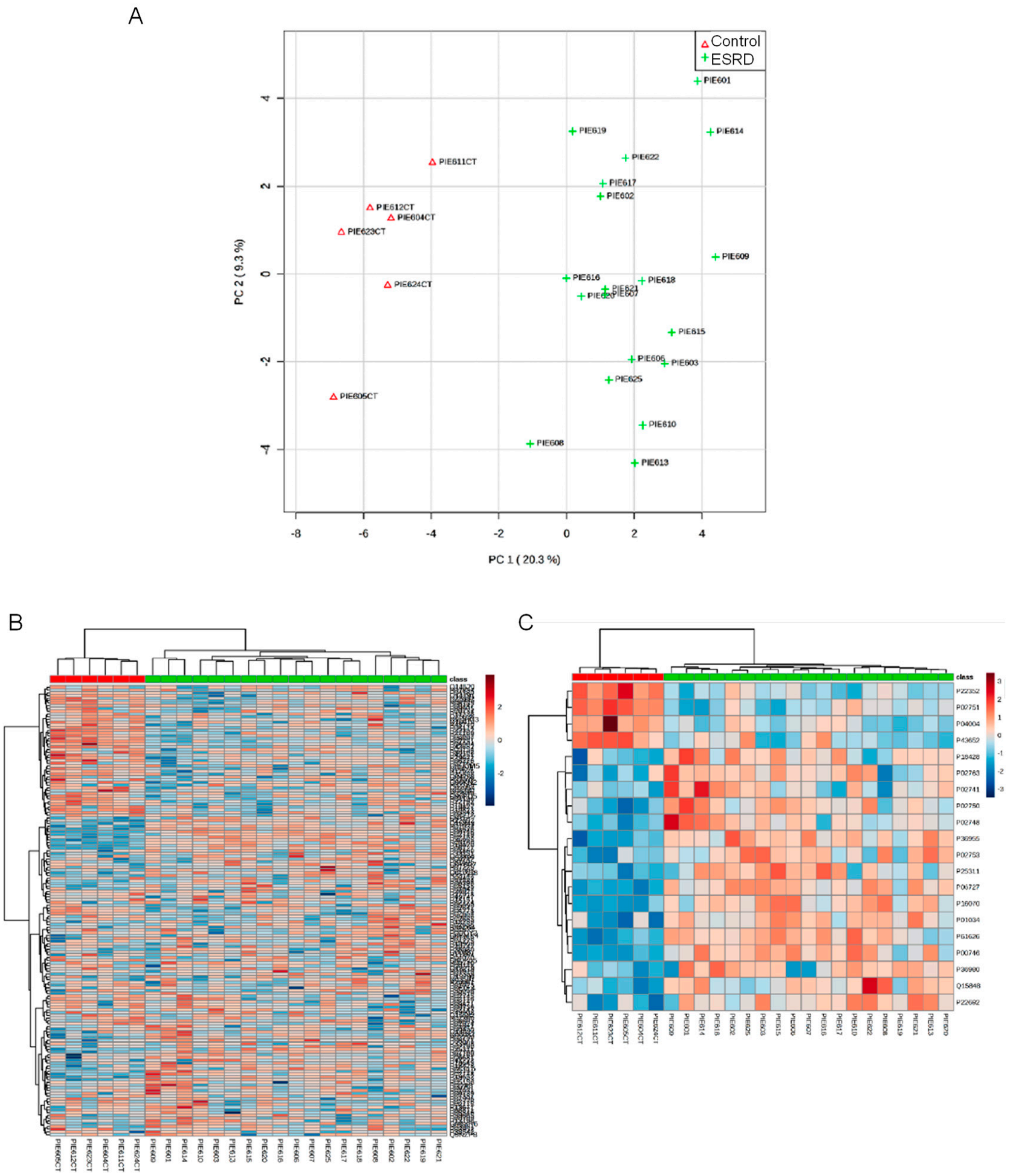

Figure S2. Unsupervised analysis from proteomics datasets. (**A**) PCA scores plot between components 1 and 2 for aCKD group. The explained variances are shown in brackets. (**B**) Unsupervised hierarchical clustering heatmap on 164 proteins for all samples in aCKD patients (green) and 6 controls (red). (**C**) Unsupervised hierarchical clustering heatmap based on the top 20 proteins selected from PLS-DA VIP. In B and C plots, a z-score transformation was performed on the intensity of each protein across all samples and each sample z-score is displayed in the heatmap. Proteins and samples were clustered by Euclidean distance and Ward linkage.

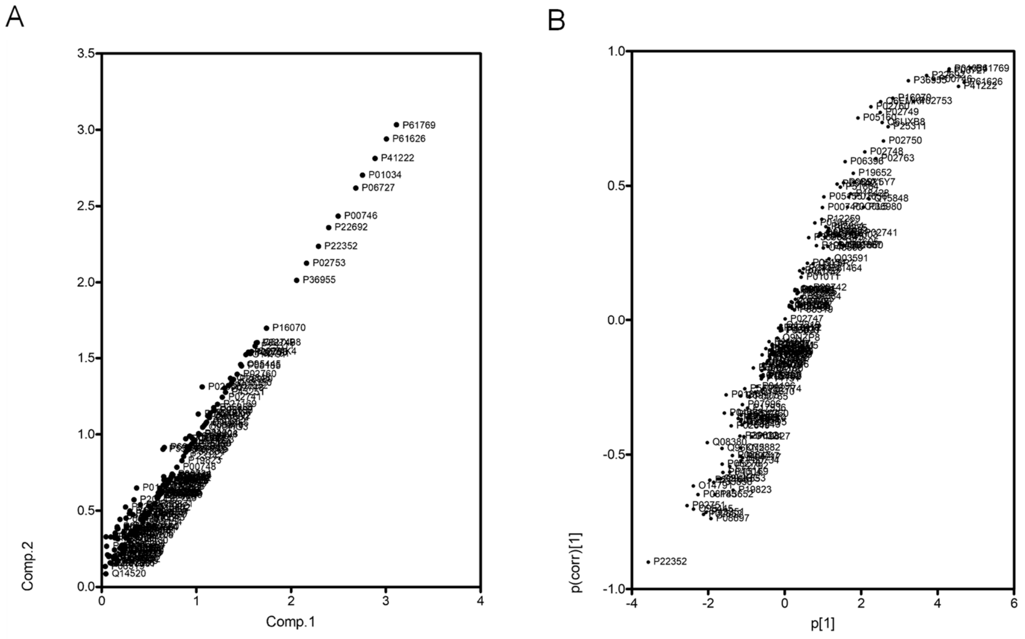

Figure S3. Supervised analysis from proteomics datasets. (**A**) PLS-DA loading plot for the aCKD group showing the relative contribution of each component to class separation. (**B**) OPLS-DA loading S-plot for the aCKD group showing the relative contribution of each variable to class separation. Each dot corresponds to one protein. The p(corr)[1] axis represents the correlation of the variable towards class separation, whereas the p[1] axis represents the magnitude of the variable.

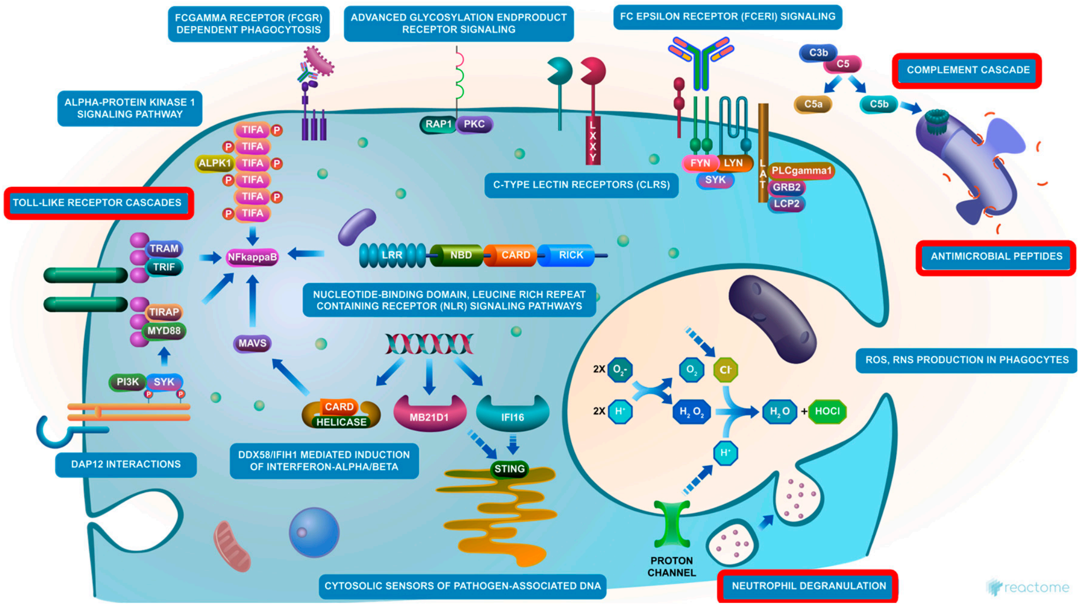

Figure S4. Altered elements from innate immune system (HSA-168249) in aCKD patients. Pathways altered in aCKD patients are highlighted in red. Proteins altered are involved in the following pathways; *Complement cascade*: CPN1,CPN2, C3, C4B, C4BPA, C8G, C9, CPB2, SERPING1, VTN, CFD, F2; *Neutrophil degranulation*: ORM1, ORM2, LRG1, CST3, CFD, LYZ, CD44, GSN, LBP, A1BG, B2M; *Antimicrobial peptide*: PGLYRP2, LYZ; *Toll-like receptor cascade*: CD14, LBP [Reactome, 10.3180/REACT_6802.2].

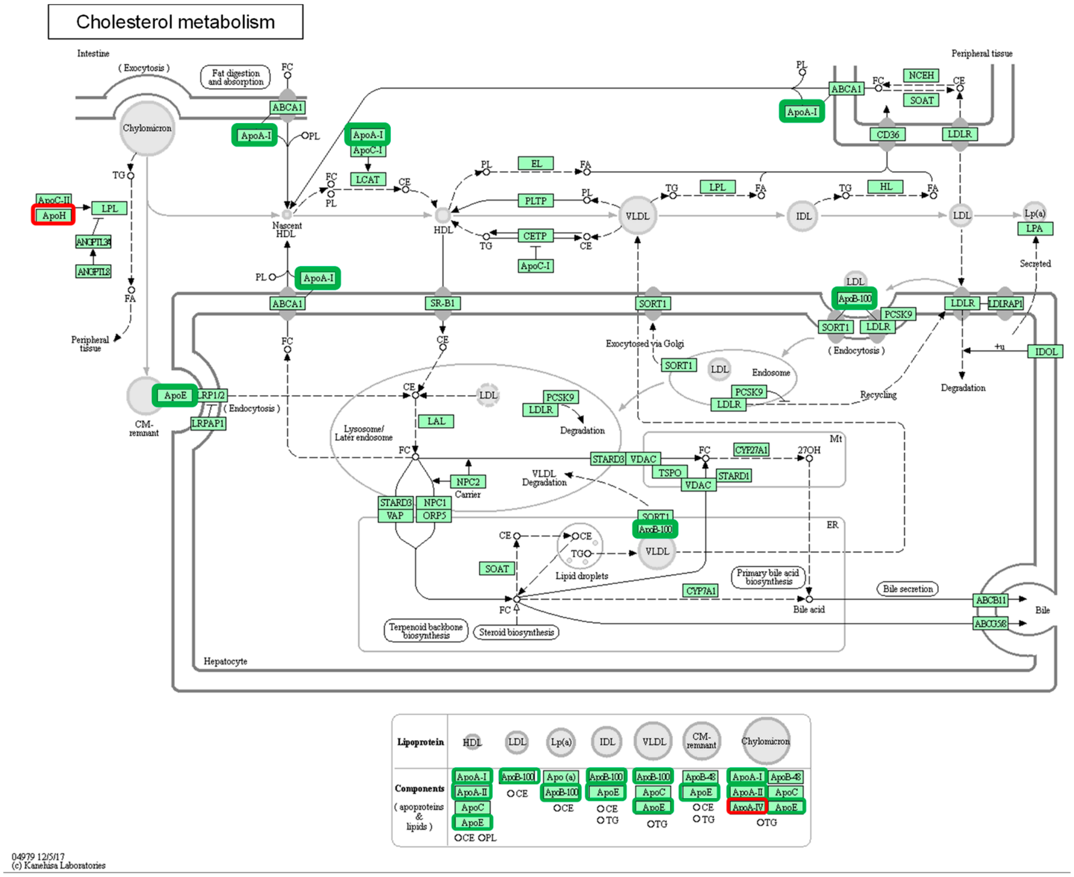

Figure S5. Altered proteins from cholesterol metabolism (KEGG–hsa04979) in aCKD patients. Proteins that showed significantly lower and higher concentrations in aCKD are shown in green and red respectively. The The 7 proteins altered are APOA1, APOA2, APOA4, APOB, APOC3, APOE, and APOH.

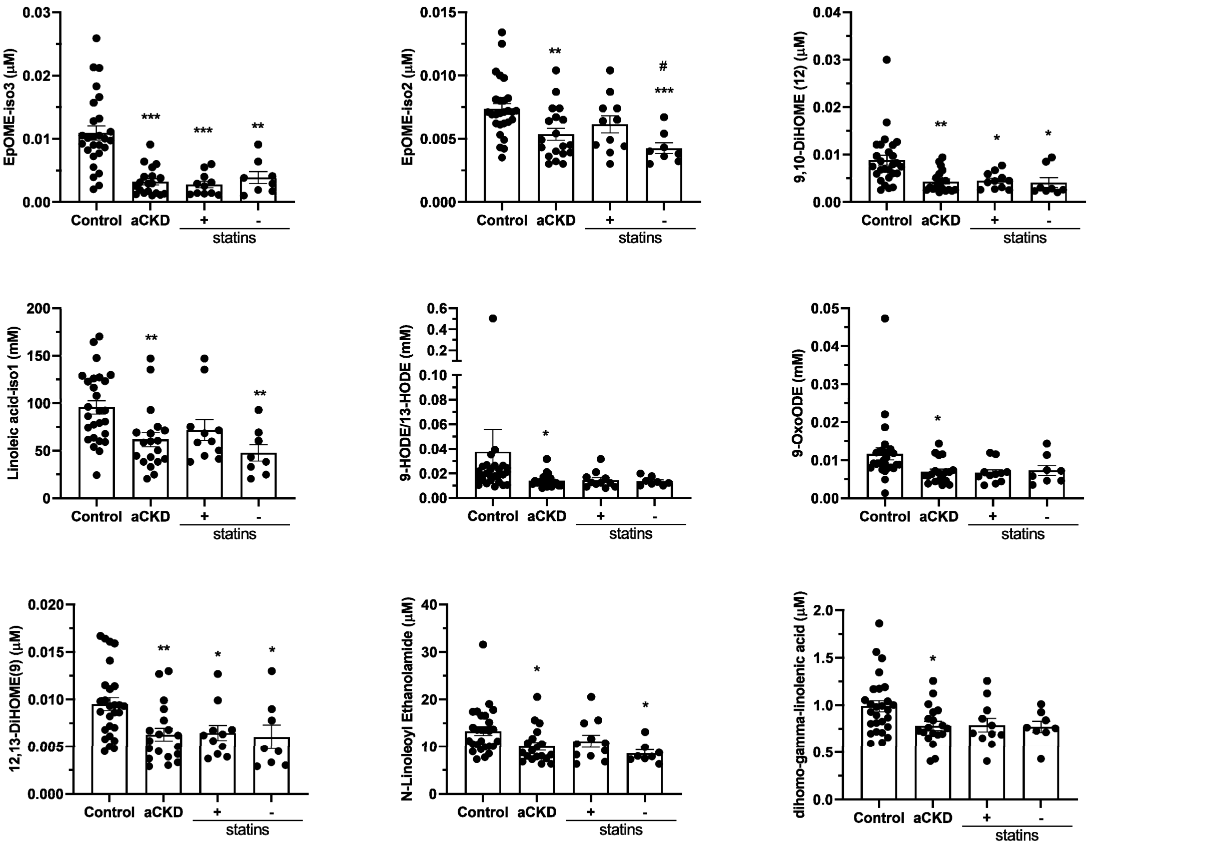

Figure S6. Impact of statins on metabolites from Linoleic acid metabolism. * Significantly different when compared to the Control group (* $p < 0.05$; ** $p < 0.01$; *** $p < 0.001$). #Significantly different when compared to aCKD patients treated with statins (# $p < 0.05$).
